# Hierarchical Self-Assembly of Thickness-Modulated Block Copolymer Thin Films for Controlling Nanodomain Orientations inside Bare Silicon Trenches

**DOI:** 10.3390/polym13040553

**Published:** 2021-02-13

**Authors:** Jin Yong Shin, Young Taek Oh, Simon Kim, Hoe Yeon Lim, Bom Lee, Young Chun Ko, Shin Park, Seung Won Seon, Se Gi Lee, Seung Soo Mun, Bong Hoon Kim

**Affiliations:** 1 Department of Organic Materials and Fiber Engineering, Soongsil University 369 Sangdo-ro, Dongjak-gu, Seoul 06978, Korea; thankusin@gmail.com (J.Y.S.); kimsimon9410@gmail.com (S.K.); tlsrlfn104@gmail.com (H.Y.L.); lovbom0110@gmail.com (B.L.); asdf082560@gmail.com (Y.C.K.); lsg6957@soongsil.ac.kr (S.G.L.); ssmun1004@gmail.com (S.S.M.); 2 Department of Smart Wearable Engineering, Soongsil University 369 Sangdo-ro, Dongjak-gu, Seoul 06978, Korea; oyti0515@gmail.com (Y.T.O.); parkshin94@gmail.com (S.P.); ssun@soongsil.ac.kr (S.W.S.)

**Keywords:** block copolymers, thickness gradient, directed self-assembly, graphoepitaxy, defect, nanolithography

## Abstract

We study the orientation and ordering of nanodomains of a thickness-modulated lamellar block copolymer (BCP) thin film at each thickness region inside a topological nano/micropattern of bare silicon wafers without chemical pretreatments. With precise control of the thickness gradient of a BCP thin film and the width of a bare silicon trench, we successfully demonstrate (i) perfectly oriented lamellar nanodomains, (ii) pseudocylindrical nanopatterns as periodically aligned defects from the lamellar BCP thin film, and (iii) half-cylindrical nanostructure arrays leveraged by a trench sidewall with the strong preferential wetting of the PMMA block of the BCP. Our strategy is simple, efficient, and has an advantage in fabricating diverse nanopatterns simultaneously compared to conventional BCP lithography utilizing chemical pretreatments, such as a polymer brush or a self-assembled monolayer (SAM). The proposed self-assembly nanopatterning process can be used in energy devices and biodevices requiring various nanopatterns on the same device and as next-generation nanofabrication processes with minimized fabrication steps for low-cost manufacturing techniques.

## 1. Introduction

Directed self-assembly (DSA) lithography based on a block copolymer (BCP) can be used to fabricate nanometer-scale patterns on a large scale; therefore, it can be applied to various nanomaterials and nanodevices [1,2,3]. One of the major research streams of BCP-based DSA is to design perfectly oriented nanodomains of BCP thin films. In particular, chemical epitaxy [4,5], graphoepitaxy [6,7,8], electric/magnetic field [9,10], mechanical shear [11], and solvent/laser annealing [12,13] are representative examples in the research field of BCP lithography. Among these techniques, graphoepitaxy using topological confinement is a promising next-generation semiconductor device fabrication process [14]. In general, however, the technique requires a thermal annealing process that requires either chemical pretreatments such as a polymer brush/self-assembled monolayer (SAM) or a solvent annealing process that takes a significant time [15,16,17]. Considering that fabrication techniques with either complicated steps or long lead times are not likely to be selected as next-generation nanopatterning solutions, the graphoepitaxy technique requires improvement. Moreover, in the graphoepitaxy approach, the primary goal is to perfectly align the surface perpendicular to the BCP nanodomain; therefore, realizing both the surface-perpendicular and surface-parallel BCP nanodomains simultaneously on the single substrate is challenging [18,19,20]. Fabricating such morphology is possible with control of the local surface energy of the substrate; however, this requires additional nanopatterning processes such as lithography and dry etching.

In this research, we propose a novel DSA process that can perfectly align lamellar BCP nanodomains with various orientations without chemical pretreatments based on a thickness-modulated BCP thin film with the graphoepitaxy method. First, we fabricate a BCP thin film with a thickness gradient in a bare silicon trench. By controlling both the effect of (1) generating the surface-parallel BCP nanodomains on the sidewall or bottom of a hydrophilic trench and (2) aligning lamellar BCP nanodomains along the thickness-gradient direction simultaneously, BCP nanodomains oriented in different directions are successfully fabricated on the same substrate. Moreover, we realize (i) perfectly oriented lamellar BCP nanodomains, (ii) pseudocylindrical nanopatterns composed of periodically aligned defects from a lamellar BCP thin film, and (iii) half-cylindrical nanostructure arrays leveraged by a trench sidewall with the strong preferential wetting of one block of the BCP by decreasing the trench width. Our novel BCP lithography technique is simple and composed of minimal processing steps; therefore, it is a DSA technique that can be applied to diverse electronic and optoelectronic, energy, and biomedical devices.

## 2. Materials and Methods

### 2.1. Silicon Trench Pattern Preparation

The conventional photolithography process for silicon trench fabrication was performed by spin-coating a positive-tone photoresist (AZ 5214) on an 8-inch-sized silicon wafer (Track System K-Spin8, DNS KOREA CO, Suwon, Korea). Soft baking was then performed to evaporate the residual solvent and densify the photoresist film. The KrF source (KrF scanner, NSR-S203B, wavelength = 284.4 nm, Nikon INC, Tokyo, Japan) was exposed to a photoresist film through a pattern mask, and the unexposed part of the resist to the KrF source was selectively crosslinked by postbaking. The exposed photoresist film was immersed in the developer for 60 s. Subsequently, a nano/micropatterned silicon trench was prepared by successive reactive ion etching (RIE) using SF_6_ and C_4_F_8_ gas (1000 W, 50 sccm, 30 s). The residual photoresist was removed by O_2_ plasma treatment (50 W, 100 sccm, 5 min) after the RIE process. 

### 2.2. Directed Self-Assembly of Curvature Block Copolymer Films

PS-*b*-PMMA (Polymer Source Inc, Quebec, QC, Canada), with molecular weights of 25–26, 44–45, and 105–106 kg/mol, was used in this work. Before the spin-coating process, the silicon wafer was washed with IPA and acetone under sonication for 30 min, and O_2_ plasma cleaning (Covance, Femto Science, Seoul, Korea) was performed for 5 min. BCP thin films were spin-coated from a 1–2.5 wt% toluene solution (Sigma-Aldrich Inc., St. Louis, MO, USA) on topological nano/micropatterned substrates. BCP thin films were then thermally annealed in a vacuum oven at 250 °C for 24 h (Figure S1). Afterwards, a PMMA block of the BCP thin film was selectively removed by a reactive ion etching (RIE) process with O_2_ plasma (50 W, 50 sccm, 15 s) (Scientech Inc, Bucheon, South Korea) for SEM characterization.

### 2.3. Characterization

The nanoscale morphology of the BCP thin film was characterized by a field-emission scanning electron microscope (FE-SEM, Gemini SEM 300, Carl Zeiss Microscopy GmbH, Oberkochen, Germany). When conducting the SEM analysis, samples were coated with a Pt sputter (Q150RS, Quorum, East Susse, UK) to eliminate the charging effect by the accumulation of static electric charges on the surface of the polymer sample. The orientations and periods of the BCP nanodomains were analyzed by the ImageJ software (Figure S2, National Institutes of Health, Bethesda, MD, USA).

## 3. Results and Discussion

**Figure 1:**
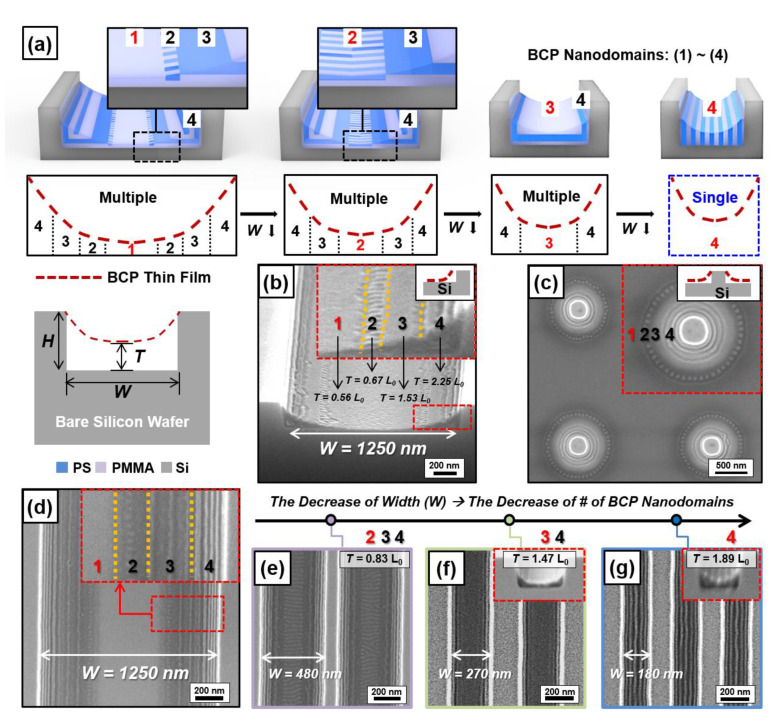
Evolution of the multiple orientations of a lamella-forming block copolymer (BCP) nanodomain inside a bare silicon trench with different widths. (**a**) Schematic illustration of the evolution of multiple orientations of lamella-forming BCP nanodomains. Top-view SEM images of BCP (PS-*b*-PMMA, M_n_ = 44–45 kg/mol) nanodomains (**b**) inside a silicon trench (W = 1250 nm) and (**c**) silicon pillar array. (**d**–**g**) Top-view SEM images of the evolution of multiple orientations of lamella-forming BCP nanodomains (PS-*b*-PMMA, M_n_ = 25–26 kg/mol) inside a silicon trench (W = 180–1250 nm).

We fabricated a thickness-modulated lamella-forming PS-*b*-PMMA thin film on a silicon substrate with periodic topographic confinement. It is noteworthy that the used bare silicon substrate is free of chemical pretreatments [21,22,23,24]. Unlike a lamella-forming BCP thin film, where chemical pretreatment such as a polymer brush is needed to realize the surface-perpendicular orientation, we simplified the fabrication process by using a bare silicon wafer without a neutral layer [25]. Figure 1a shows the evolution of the multiple orientations of BCP nanodomains in a thickness-modulated lamella-forming PS-*b*-PMMA thin film when the height (H) of the silicon trench is 194 nm and its width (W) is decreased. There are two significant effects. (a) Firstly, there is a tendency to form BCP nanodomains with a parallel orientation to the sidewall or bottom surface of the silicon trench. This is because every surface of the silicon trench is hydrophilic as there is no chemical pretreatment. For example, there is a stronger tendency to form BCP nanodomains with a parallel orientation to the sidewall of the silicon trench as it is close to the edge of the silicon trench. At the center of the silicon trench, this tendency is minimal, and a BCP nanodomain with a parallel orientation to the bottom surface of the silicon trench is formed. (b) Secondly, a thickness-modulated BCP lamella tends to align along the thickness-gradient direction, and the tendency becomes stronger when the thickness gradient is steeper [26,27]. Figure 1b represents the four different types of orientations of BCP nanodomains when there is a thickness-gradient BCP thin film in the wide trench (W = 1250 nm). If we symbolize the bottom-surface-parallel orientation and the sidewall-parallel orientation as L ═ and L║, we can express the four different orientations of the BCP nanodomains from the center of the silicon trench to the sidewall as L ═ → L║ → L ═ → L║. Such orientations were also found from the silicon pillar (Figure 1c). When we reduced the silicon trench width (W), it was found that the number of multiple nanodomains was decreased, and the effect of the sidewall of the silicon trench on the BCP nanodomain was increased. For example, a thickness-modulated PS-*b*-PMMA (M_n_ = 25–26 kg/mol) thin film contains four, three, two, and one nanodomain when W = 1250, 480, 270, and 180 nm, respectively. Each nanodomain showed different characteristics and is summarized below. Briefly, four kinds of BCP nanodomains (Nanodomains (1)–(4)) were found at the center of the silicon trench when W = 1250, 480, 270, and 180 nm, respectively.

*Nanodomain (1): Because it is located in the most distant location from the sidewall of the silicon trench, a BCP nanodomain parallel to the bottom surface of the silicon trench is formed (Figure 1d). This domain is the thinnest part of a thickness-modulated BCP thin film, and its thickness is almost uniform. Additionally, we confirm that the PMMA block has a higher tendency of wetting the sidewall or bottom surface of the silicon trench than the PS block using the harmonic mean equation [28].*

*Nanodomain (2): When W = 480 nm, there are tendencies of forming BCP nanodomains both parallel to the sidewall (L║) and bottom surface (L═) (Figure 1e). As a result, a T-junction tilt boundary of the BCP nanodomain [29,30,31] is formed and well-aligned along the thickness-gradient direction (T = 0.83 L_0_, L_0_ = periodicity of the lamellar structure)*.

*Nanodomain (3): A BCP nanodomain parallel to the bottom surface (L═) is formed (T = 1.47 L_0_, Figure 1f).*

*Nanodomain (4): A BCP nanodomain strongly tends to align along with the parallel direction of the sidewall (L║), and there is only one orientation in the entire BCP thin film (T = 1.89 L_0_, Figure 1g). This domain corresponds to the thickest part, and its thickness is almost uniform.*

In addition, we can confirm that the evolution of the BCP nanodomain orientation according to the width (W) changes of the silicon trench appears to be the same, even if the molecular weight (M_n_) of the lamella-forming BCP is changed. For example, the BCP nanodomain (L═) parallel to the bottom surface has a height of 0.56 L_0_ or 1.53 L_0_ with the PS-*b*-PMMA thin film (M_n_ = 45–44 kg/mol, Figure 1b and Figure S3). Interestingly, the BCP thin film has a single orientation of the nanodomain when the width of the silicon trench becomes narrow enough (e.g., W = 180 nm) so that the effect of the sidewall of the silicon trench on the BCP thin film is maximized.

**Figure 2:**
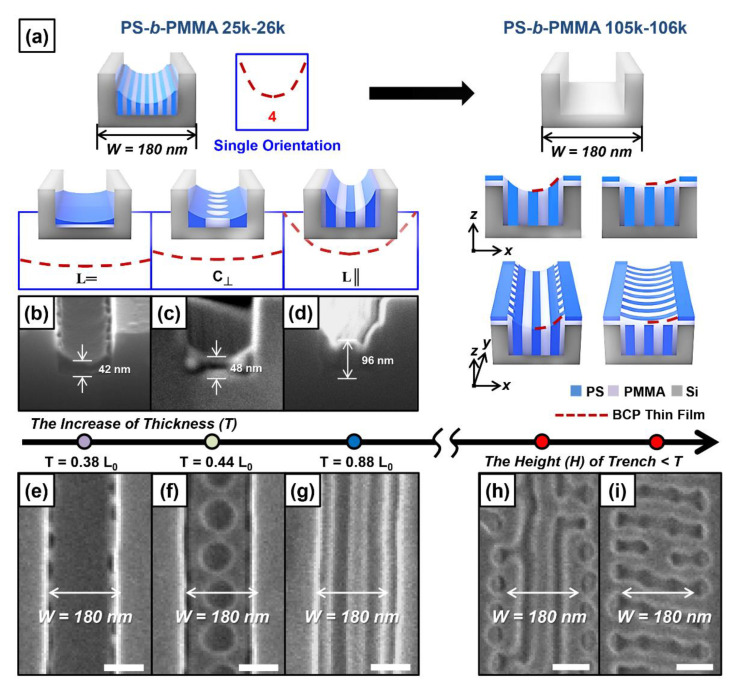
Single orientation of a lamella-forming BCP (PS-*b*-PMMA, M_n_ = 105–106 kg/mol, L_0_ = 109 nm) thin film inside a silicon trench (scale bar = 100 nm). (**a**) Schematic illustration of various single orientations of lamella-forming BCP thin films. Cross-view (**b–d**) and top-view (**e**–**i**) SEM images of BCP thin films inside a silicon trench (T = 42, 48, 96, 244, and 274 nm).

We also investigated the changes of the orientation of the BCP nanodomain according to the thickness (T) changes of the BCP thin film under the condition of having large effects of the sidewall of the silicon trench on the BCP thin film. For this purpose, we fabricated a large-molecular-weight lamellae-forming PS-*b*-PMMA thin film (M_n_ = 105–106 kg/mol) with a thickness gradient in the bare silicon trench using the spin-coating method (Figure 2a). Afterwards, we prepared BCP thin films with various thicknesses (T) in the same trench width (W = 180 nm) and monitored the orientation of the BCP nanodomain. Figure 2b,d,e,g shows tilted-view and top-view SEM images of a lamella-forming PS-*b*-PMMA thin film (M_n_ = 105–106 kg/mol) under the confinement of a bare silicon trench (W = 180 nm) with an increased thickness (T) of the BCP thin film, respectively.

(1) When T = 42 nm, the BCP thin film has a bottom-surface-parallel orientation (L═) due to the low thickness. In this configuration, the sidewall of the silicon trench cannot affect the orientation of the BCP nanodomain (Figure 2b,e).
(2) When T = 48 nm, the BCP nanostructure analogous to the perforated layer is formed (Figure 2c,f). Considering the period of the BCP lamellar nanostructure, this is a metastable nanostructure, the cylindrical defects of which are periodically trapped due to an extremely narrow silicon trench width and the low mobility of the polymer chain caused by the large molecular weight and low thickness (T) of the BCP thin film. We named this morphology, which is similar to the bottom-surface-perpendicular cylinder, a pseudocylindrical nanodomain (**C_丄_**).
(3) When T = 96 nm, the effect of the sidewall of the silicon trench becomes larger due to the high thickness. Therefore, a BCP nanodomain with a sidewall-parallel orientation is formed (Figure 2d,g).
(4) There are two orthogonally oriented lamellar nanodomains when the BCP thin film thickness (T) is higher than the silicon trench height (H = 194 nm) (Figure 2h,i) [32]. This is a typical nanostructure that minimizes the total interfacial energy of the BCP nanodomain, and the orientation of the upper lamellar layer varies depending on the thickness (T = 244 and 274 nm in Figure 2h,i, respectively) of the BCP thin film [33,34].

**Figure 3:**
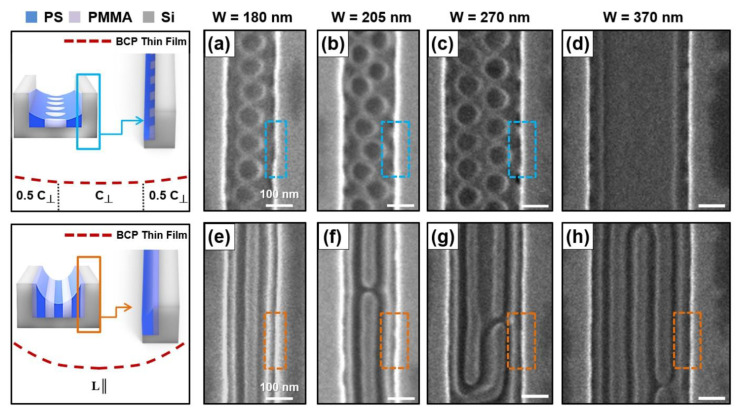
Top-view SEM images of BCP (PS-*b*-PMMA, M_n_ = 105–106 kg/mol, L_0_ = 109 nm) nanodomains with a 48- (**a**–**c**), 15- (**d**), and 96-nm-thick (**e**–**h**) thin film inside various silicon trenches.

Figure 3 represents the BCP nanostructure in the vicinity of the silicon trench sidewall when a high-molecular-weight PS-*b*-PMMA thin film (M_n_ = 105–106 kg/mol) with a thickness gradient is prepared with various widths (W) inside the bare silicon trench. Figure 3a–c presents top-view SEM images of a 48-nm-thick PS-*b*-PMMA thin film inside a silicon trench. In this case, due to the metastable nanostructure, the bottom-surface-perpendicular half-cylindrical defects (0.5 **C_丄_**) are periodically trapped near the trench sidewall. When the silicon trench width (W) becomes 370 nm, the effect of the trench sidewall on the BCP thin film is diminished; therefore, a bottom-surface-parallel BCP nanodomain (L═) is observed (Figure 3d). Figure 3e–h shows the formation of the BCP nanodomain with a sidewall-parallel orientation near the trench sidewall when a 96-nm-thick PS-*b*-PMMA thin film is present inside the silicon trench (width of 180–370 nm). We can understand the configuration in the same way as the case of the Nanodomain (4) region (Figure 1g) of the PS-*b*-PMMA thin film (M_n_ = 25–26 kg/mol).

**Figure 4:**
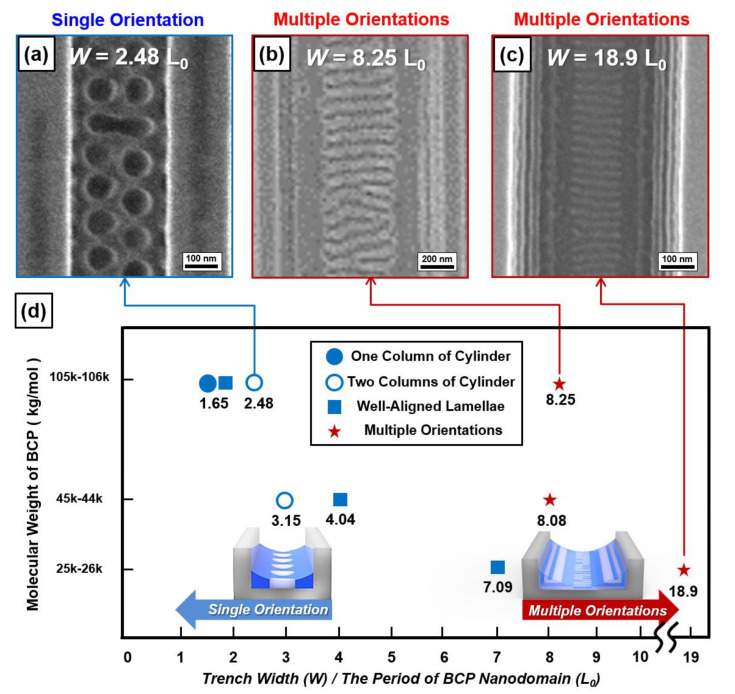
Top-view SEM images of lamella-forming BCP nanodomains inside various silicon trenches when (**a**) W = 2.48 L_0_, (**b**) W = 8.25 L_0_, and (**c**) W = 18.9 L_0_. (**d**) Morphology mapping for PS-*b*-PMMA (M_n_ = 105–106, 44–45, and 25–26 kg/mol) nanodomains inside various silicon trenches.

In addition, we investigated the conditions that determine the number of BCP nanodomain orientations by varying the BCP molecular weight (M_n_) and width (W) of the bare silicon trench. For example, Figure 4a shows the bottom-surface-perpendicular pseudocylindrical nanodomain (**C_丄_**) with a single orientation when M_n_ of the PS-*b*-PMMA thin film is 105–106 kg/mol and the silicon trench width (W) is 2.48 times the BCP periodicity (L_o_). Figure 4b,c shows the multiple orientations of the BCP nanodomain when M_n_ is 105–106 and 25–26 kg/mol and the silicon trench width (W) is 8.25 and 18.9 times the BCP periodicity (L_o_), respectively. The formed multiple orientations of the BCP nanodomain are found to have a similar morphology to Nanodomain (2) in Figure 1a. We summarize whether the PS-*b*-PMMA thin film with a wide range of M_n_ (105–106, 44–45, and 25–26 kg/mol) inside the silicon trench with different widths (W) forms a single orientation or not in Figure 4d. The results reveal that a single orientation of the BCP nanodomain is formed when the silicon trench width (W) is smaller than 4.04 L_o_ (Figure S4).

**Figure 5:**
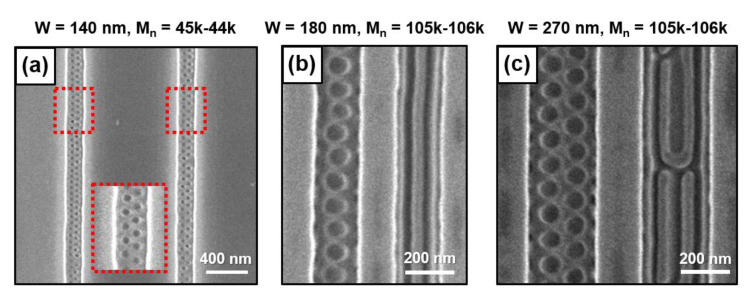
(**a**) Top-view SEM image of the surface-perpendicular pseudocylindrical nanodomain (**C_丄_**) on the trench area and the surface-parallel lamellar nanodomain on the mesa area (PS-*b*-PMMA, 44–45 kg/mol, W = 140 nm). (**b**,**c**) Surface-perpendicular pseudocylindrical nanodomain (**C_丄_**) and the sidewall-parallel lamellar nanodomain (L║) in the trench area (PS-*b*-PMMA, 105–106 kg/mol, W = 180 and 270 nm).

In the conventional BCP graphoepitaxy process, chemical pretreatments produce uniform surface energy in the entire substrate. Accordingly, fabricating the surface-perpendicular and surface-parallel BCP nanodomain simultaneously is challenging unless a nano/micropatterning process is added to change the surface energy of a specific part of the substrate. Figure 5a shows a top-view SEM image of the surface-perpendicular pseudocylindrical nanodomain (**C_丄_**) on the trench area and the surface-parallel lamellar nanodomain on the mesa area, using a bare silicon trench (W = 140 nm) and a lamella-forming PS-*b*-PMMA thin film (44–45 kg/mol). Figure 5b,c shows that the fabrication of the surface-perpendicular pseudocylindrical nanodomain (**C_丄_**, T = 48 nm) and the sidewall-parallel lamellar nanodomain (L║, T = 96 nm) in the trench area at once when we carefully chose the width of the silicon trench and the thickness of the BCP thin film.

## 4. Conclusions

In summary, we studied the morphology of nanodomains of a BCP thin film with a thickness gradient in the topological nano/micropattern of a bare silicon trench without chemical pretreatments. Our study reveals that the morphology of the BCP nanodomain is strongly affected by two factors: (a) the tendency of wetting the PMMA block parallel to the bottom surface or sidewall of the bare silicon trench and (b) the tendency of aligning lamella-forming BCP nanodomain along the thickness gradient of the BCP thin film. Additionally, we carefully controlled the width of a bare silicon trench and a thickness (T) of BCP thin film to fabricate (i) perfectly oriented lamellar nanodomains, (ii) a pseudocylindrical nanodomain as periodically aligned defects from the lamellar BCP thin film, and (iii) half-cylindrical nanostructure arrays leveraged by a trench sidewall with the strong preferential wetting of one block of the BCP in a large area. In particular, we found that a single orientation of the BCP nanodomain can be formed when the width of the bare silicon trench is very narrow, and the BCP nanodomain has a single orientation when the ratio of the width (W) of the bare silicon trench to the periodicity (L_0_) of the BCP nanostructure is smaller than 7.09.

Our approach has the following advantages: (1) a complicated step for tuning the substrate surface energy is not necessary because the BCP thin film is fabricated on the bare silicon trench. Considering that the self-assembly-based nanofabrication process requires a long processing time, reducing a processing step can be an important advantage. (2) A nanohole/line array can be fabricated on the same substrate simultaneously because a single type of BCP forms different nanostructures depending on its film thickness (T). (3) Multiple orientations of the BCP nanodomain can be fabricated on a single substrate as not only the BCP nanodomain’s vertical/parallel orientation but a direction of lateral ordering can be controlled. Our novel BCP graphoepitaxy process can be applicable to the large-scale production of next-generation electronic, optoelectronic, and energy devices that require various nanometer-scale patterns [35,36,37,38,39].

## Data Availability

No new data were created or analyzed in this study. Data sharing is not applicable to this article.

## Supplementary Materials

The following are available online at https://www.mdpi.com/2073-4360/13/4/553/s1, Figure S1. (**a**) The schematic illustration of the directed self-assembly (DSA) process of a thickness-modulated block copolymer (BCP) thin film inside bare silicon trenches. Cross-sectional SEM images of (**b**) silicon trenches and (**c**–**d**) thickness-modulated BCP thin films, Figure S2. Top-view SEM images of lamella-forming PS-*b*-PMMA nanodomains on a chemically neutral silicon substrate: (**a**) 25–26, (**b**) 44–45, and (**c**) 105–106 kg/mol, Figure S3. Tilted-view SEM images of the PS-*b*-PMMA thin film (44–45 kg/mol) inside a silicon trench: (**a**) W = 1250 nm, (**b**) W = 360 nm, (**c**) W = 270 nm, and (**d**) W = 225 nm, Table S1. Single/multiple orientations of lamella-forming BCP thin films inside various silicon trenches.
