# Supplementary material for: Hierarchical Self-Assembly of Thickness-Modulated Block Copolymer Thin Films for Controlling Nanodomain Orientations inside Bare Silicon Trenches
Source: Polymers (Basel). 2021 Feb 13;13(4):553. doi: 10.3390/polym13040553 (PMC7918743; doi:10.3390/polym13040553)
Supplement: Supplementary file 1 [file polymers-13-00553-s001.pdf]

polymers

MDPI

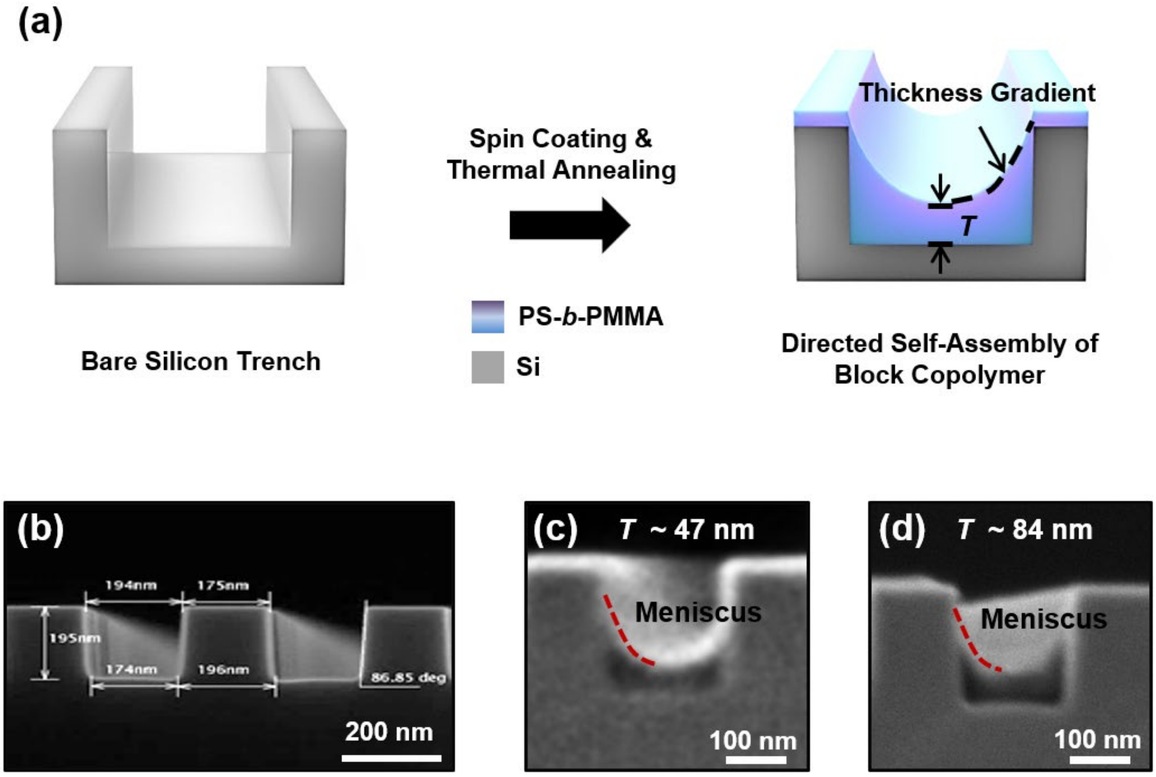

Figure S1. (a) The schematic illustration of directed self-assembly (DSA) process of a thickness-modulated block copolymer (BCP) thin film inside bare silicon trenches. The cross-sectional SEM images of **(b)** silicon trenches and **(c)** & **(d)** thickness-modulated BCP thin films.

M_n of BCP	PS-*b*-PMMA 25k-26k	PS-*b*-PMMA 44k-45k	PS-*b*-PMMA 105k-106k
L_0 (nm)	25.4	44.5	109

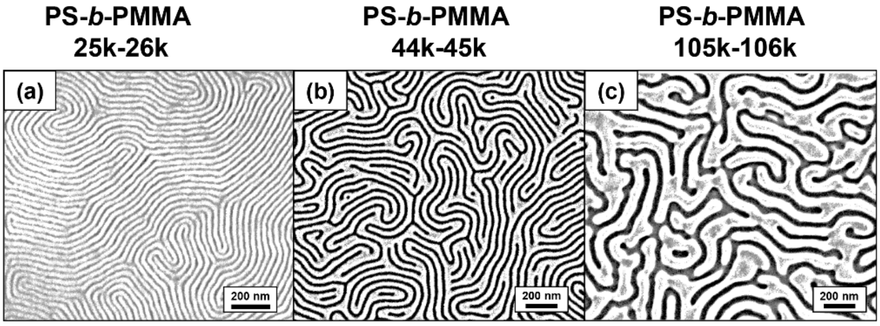

Figure S2. Top view SEM images of lamella-forming PS-*b*-PMMA nanodomains on chemically neutral silicon substrate. (a) 25 kg/mol – 26 kg/mol, (b) 44 kg/mol – 45 kg/mol, and (c) 105 kg/mol – 106 kg/mol.

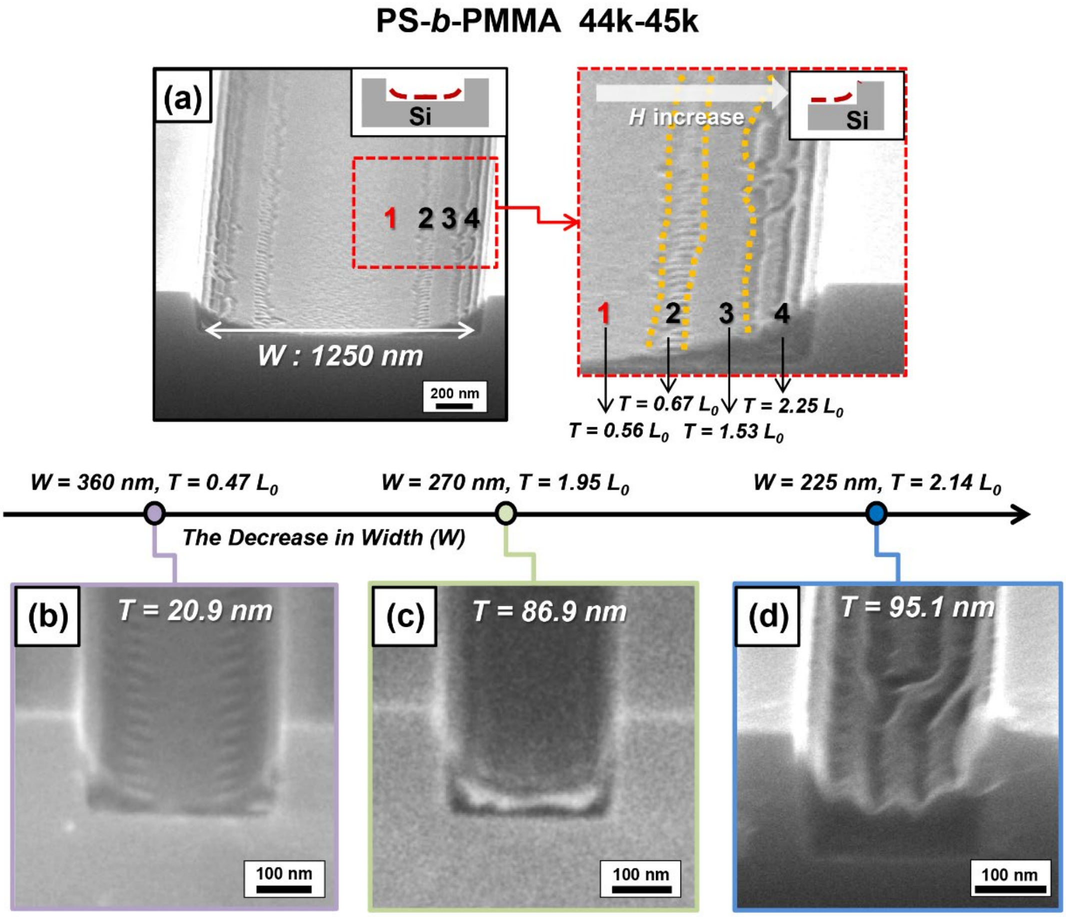

Figure S3. The tilted-view SEM images of PS-*b*-PMMA thin film (44 kg/mol – 45 kg/mol) inside silicon trenches. **(a)** W = 1250 nm, **(b)** W = 360 nm, **(c)** W = 270 nm, and **(d)** W = 225 nm.

Table S1. Single/multiple orientations of lamella-forming BCP thin films inside various silicon trenches.

BCP	W_{Trench} (nm)	T_{BCP} (nm)	Orientation
PS-*b*-PMMA 25k-26k (L_0 = 25.4 nm)	W = 49.21 L_0	T = 0.63 L_0	Multiple
	W = 18.9 L_0	T = 0.83 L_0	Multiple
	W = 10.62 L_0	T = 1.47 L_0	Multiple
	W = 7.09 L_0	T = 1.89 L_0	Single [$L\parallel$]
PS-*b*-PMMA 44k-45k (L_0 = 44.5 nm)	W = 28.09 L_0	T = 0.27 L_0	Multiple
	W = 8.09 L_0	T = 0.47 L_0	Multiple
	W = 6.07 L_0	T = 1.95 L_0	Multiple
	W = 4.04 L_0	T = 2.14 L_0	Single [$L\parallel$]
PS-*b*-SMMA 105k-106k (L_0 = 109 nm)	W = 1.65 L_0	T = 0.38 L_0	Single [$L=$]
	W = 1.65 L_0	T = 0.44 L_0	Single [$C\perp$]
	W = 1.65 L_0	T = 0.88 L_0	Single [$L\parallel$]
